# Identifying Environmental Risk Factors for Louping Ill Virus Seroprevalence in Sheep and the Potential to Inform Wildlife Management Policy

**DOI:** 10.3389/fvets.2020.00377

**Published:** 2020-06-30

**Authors:** Lucy Gilbert, Franz Brülisauer, Kim Willoughby, Chris Cousens

**Affiliations:** ^1^Institute of Biodiversity, Animal Health and Comparative Medicine, University of Glasgow, Glasgow, United Kingdom; ^2^Scotland's Rural College (SRUC), Edinburgh, United Kingdom; ^3^Moredun Research Institute, Pentlands Science Park, Penicuik, United Kingdom

**Keywords:** deer, ticks, GIS, habitat management, *Ixodes ricinus*, tick-borne disease

## Abstract

Identifying the risk factors for disease is crucial for developing policy and strategies for controlling exposure to pathogens. However, this is often challenging, especially in complex disease systems, such as vector-borne diseases with multiple hosts and other environmental drivers. Here we combine seroprevalence data with GIS-based environmental variables to identify the environmental risk factors associated with an endemic tick-borne pathogen—louping ill virus—in sheep in Scotland. Higher seroprevalences were associated with (i) upland/moorland habitats, in accordance with what we predicted from the habitat preferences of alternative LIV transmission hosts (such as red grouse), (ii) areas of higher deer density, which supports predictions from previous theoretical models, since deer are the key *Ixodes ricinus* tick reproduction host in this system, and (iii) a warmer climate, concurring with our current knowledge of how temperature affects tick activity and development rates. The implications for policy include adopting increased disease management and awareness in high risk habitats and in the presence of alternative LIV hosts (e.g., grouse) and tick hosts (especially deer). These results can also inform deer management policy, especially where there may be conflict between contrasting upland management objectives, for example, revenue from deer hunting vs. sheep farmers.

## Introduction

Identifying the risk factors for disease is crucial for developing policy and strategies for controlling exposure to pathogens. This is often challenging, depending on the type of disease system and the complexity of its epidemiology. The prevalence and spread of many livestock diseases are influenced primarily by the densities and movements of the livestock themselves, for example, bovine viral diarrhea (1). The risk factors become more diverse and the epidemiology more complex when the pathogen has alternative reservoir hosts in addition to the livestock, e.g., *Mycobacterium bovis* the causative agent of bovine tuberculosis in domestic cattle, which can have wildlife reservoir hosts including red deer *Cervus elaphus*, wild boar *Sus scrofa*, and European badgers *Meles meles* (2, 3). Similarly, diseases that are vector-borne can be influenced by a range of environmental factors that affect the populations of free-living vectors, e.g., geography, climate, and habitat drive the risk of liver fluke *Fasciola hepatica* infection (fasciolosis) in livestock via their effect on the vector, the mud snail *Galba truncatula* (4, 5). However, the most complex disease systems are those with both multiple vector hosts and multiple pathogen transmission hosts, which makes disease risk difficult to predict and to control, and it is challenging to tease apart the effect of the livestock themselves from wildlife or other environmental factors on disease risk. Prime examples include tick-borne pathogens such as the *Borrelia burgdorferi* sensu lato complex of bacteria that cause Lyme borreliosis and the tick-borne encephalitis complex of viruses, which includes louping ill virus (LIV). In Europe these pathogens are vectored primarily by the most ubiquitous tick in Europe, *Ixodes ricinus*, which is a generalist, parasitizing almost all terrestrial vertebrates. It spends the vast majority of its lifecycle away from its hosts, so its survival and activity is influenced by a multitude of environmental factors [e.g., (6–10)]. This makes identifying the environmental risk factors for such pathogens extremely challenging. For example, the *B. burgdorferi* s.l. complex is transmitted by a huge range of terrestrial vertebrates including birds, rodents, hedgehogs and sheep, but not deer. Unsurprisingly, even after multiple studies, there is still no uniform consensus on how the key players in the transmission cycle interact to drive disease risk to humans. LIV is much less studied than *B. burgdorferi* s.l., and, while not as complex a system, still has multiple transmission hosts including birds and mammals. It causes illness and death in livestock, especially sheep *Ovies aries* (11), and in red grouse *Lagopus lagopus scoticus* (12), an economically valuable gamebird. LIV is prevalent in upland areas of the British Isles, and parts of Norway, Denmark and Spain [reviewed by (13)]. A national scale analysis of environmental risk factors for LIV infection in sheep has not, until now, been conducted.

Sheep can be reservoirs of LIV without the need for any other transmission hosts of tick or LIV (14–16) since sheep are competent transmission hosts (17) and also feed all active stages (larvae, nymphs and adults) of the *I. ricinus* vector (18). Therefore, it may seem reasonable to predict that higher prevalences of LIV occur in areas with higher densities of sheep. However, because *I. ricinus* feed on such a wide range of terrestrial vertebrates, including rodents, birds and deer, and because LIV can be transmitted by other hosts, most notably red grouse and mountain hares *Lepus timidus*, here we test the hypothesis that LIV prevalence in sheep farms is influenced by environmental factors associated with tick abundance and LIV transmission hosts. In Scotland heather moorland is the preferred habitat for LIV transmission hosts, red grouse and mountain hares, while sheep are stocked at much lower densities on moorland than on improved grassland. *I. ricinus* ticks are most abundant in areas with high deer densities (7, 10, 19, 20), and in the North-European context, where the climate is warmer (21, 22). Thus, if wildlife hosts and other abiotic factors are more important risk factors than the sheep themselves, we predict higher LIV seroprevalences in heather moorland than in lowland improved grassland and in areas with higher densities of deer and a warmer climate. Woodland habitats are often associated with higher *I. ricinus* tick densities than open habitats, due to generally higher tick host densities and mild, humid microclimate created by woodland canopies (10, 19, 23–25). Indeed, sheep tick burdens and tick densities on sheep pastures can be higher if they are closer to woodlands or have more tree cover (26). We therefore also predict higher LIV seroprevalences among sheep farms that have a higher proportion of woodland cover. We tested these predictions by combining a randomized seroprevalence survey in sheep farms across Scotland with GIS-based environmental data of each farm's location, with the purpose of identifying environmental risk factors of LIV to inform policy on potential disease mitigation measures. This is the first large-scale cross-sectional study to identify the environmental risk factors for LIV in sheep.

## Materials and Methods

### Sheep Farm Selection and Sample Collection

We conducted a national survey, using a stratified random sampling design based on Scottish Agricultural Census data to ensure random and representative sampling of sheep flocks for all regions over Scotland. Only flocks with at least 50 breeding ewes were included and, on farms that had multiple flocks, only one flock was used. Breeding ewes were chosen to get a representative sample for a location. Younger animals may not have yet seroconverted to endemic pathogens. Tups are likely to have been purchased from elsewhere, so that any seroconversion may have been due to infection picked up in a different location. The inclusion criterion of 50 sheep was chosen because holdings of <50 sheep often do not have the number of breeding ewes we required for sampling (*n* = 27). In addition, this ensured we excluded pet sheep and small-holdings or “hobby-farms,” which tend to have different management.

Study farms were initially contacted by mail and then by telephone to confirm which farms were willing to participate, resulting in a sample size of 125 sheep farms. The selection procedure for randomly assigning farms was as follows: A sampling frame of 825 sheep holdings was randomly generated as a random subset of the 2,004 agricultural census data held by the Scottish Government containing a total of approximately 14,400 Scottish sheep holdings. Of these, a spatially representative subset of 251 farms were approached, and 125 were recruited: 28 farms did not meet the selection criteria as they did not have the minimal required flock size of 50 breeding ewes; 91 eligible farms refused participation; on seven farms the flock could not be sampled for a variety of reasons. The final sample size of 125 farms was cross-stratified in line with the proportion of farms across the Scottish Animal Health administrative divisions, as follows: Central (22), North East (13), Northern Isles (14), Highlands (27), South East (18), and South West (22).

Each of the 125 farms was visited between July 2006 and August 2008 and approx. 10 mL of blood was collected from a random selection of 27 sheep per farm (except 26 sheep from 3 farms, and 28 sheep from 1 farm). This sample size allows a 95% confidence interval of <5% for estimating LIV sero-prevalence (15). Farmers were asked whether they had vaccinated the breeding against LIV and, if so, date of last vaccination. In case of inter-annual variation, such as potential LIV cycles, year was controlled for in the statistical models. However, from a study that monitored LIV sero-prevalence in sheep over multiple years there is no evidence for cyclical behavior or stochastic inter-annual variation in LIV (15).

### Determination of Positive LIV Samples

Hemagglutination-inhibiting antibody (HIA) tests were undertaken on sheep blood sera using chick red blood cells as described by Clarke and Casals (28). The LIV HIA test is the standard diagnostic test used in the UK to determine a serological response to LIV exposure. The test is known to detect antibody to closely related viruses such as TBE and Yellow a Fever which are not endemic in the UK; the LIV HIA test is not known to have cross-reactivity to any viruses endemic to the UK. Reciprocal HIA titers of at least 20 HIA units (a dilution titer of 1/20 or more) were regarded as sero-positive to LIV infection (29). However, vaccination against LIV had been administered at three of the 125 farms within the 6 months before blood sampling so for these three farms sero-positivity was assumed only for titers >1/160 which is consistent with ongoing exposure (15). One farm had vaccinated 2 years previously, which was assumed to not affect LIV assays as antibody titers from vaccination rapidly decline such that seroposivity after vaccination is typically well below 1/160 (30). For statistical analysis we used the estimated sero-prevalence (the proportion of sheep blood samples that tested positive) for each sheep flock.

### Environmental Variables

From GIS databases we extracted data on climate, habitat and tick hosts for the locations of each farm. Climate variables from a GIS database included variables relating to temperature and precipitation on a 1 km or 5 km grid (Table 1). All these were from Met Office 1971–2000 long-term averages and derived using the Hawth's Analysis Tools (32). We chose a long period of time for the climate averages to reduce the influence of outlying weather events and to enable generic inference nationally and irrespective of weather in a particular season. Hawth's Analysis Tools is an extension for ESRI's ArcGIS (specifically ArcMap) that performs spatial analysis and functions and is available to download online.

**Table 1:** Environmental variables from the GIS database that were originally considered for inclusion in the statistical model to describe louping ill virus seroprevalence.

**Variable type**	**Variable description**	**Units**	**Scale**	**Source**
Temperature	Growing degree days	days	5 km	Met office
	Growing season length	days	5 km	Met office
	Average days of air frost annually	days	1 km	Met office
	Average days of ground frost annually	days	1 km	Met office
	Average days of snow cover annually	days	1 km	Met office
	Average daily maximum temperature annually and monthly	°C	1 km	Met office
	Average daily minimum temperature annually and monthly	°C	1 km	Met office
	Average daily mean temperature annually and monthly	°C	1 km	Met office
Precipitation	Average precipitation annually and monthly	mm	1 km	Met office
	Dry days per year	days	5 km	Met office
Hosts	Sheep density—2003–2006	head km^−2^	2 km	AgCensus
	Cattle density—2003–2006	head km^−2^	2 km	AgCensus
	Red deer density—approximate average red deer density 2006, then Krigged	head km^−2^	2 km	DMG
Habitat	Heather moorland (undifferentiated heath, wet heath, dry heath)	%	25 m	Fuller et al. (31)
	Blanket bog	%	25 m	Fuller et al. (31)
	Montane	%	25 m	Fuller et al. (31)
	Bracken	%	25 m	Fuller et al. (31)
	Coarse (rough/acidic) grassland	%	25 m	Fuller et al. (31)
	Smooth and improved grassland	%	25 m	Fuller et al. (31)
	Broadleaf woodland	%	25 m	Fuller et al. (31)
	Coniferous woodland	%	25 m	Fuller et al. (31)
	Mixed woodland	%	25 m	Fuller et al. (31)
	“Woodland” = broadleaf+conifer+mixed	%	25 m	Fuller et al. (31)

*The Met Office data were based on 1971–2000 long term average climate data. Growing degree days is the number of days with daily temperature above 5°C. OS is Ordnance Survey, DCS is Deer Commission for Scotland, DMG is Deer Management Group. Habitat categories are derived from the Land Cover Map 2000 categories (31) and are a proportion of a 5 km radius area around the farmhouse location*.

Habitat data were derived from the UK Land Cover Map (2000) in a 50 m grid. They were split into the following categories according to those most commonly occurring around the farms: bracken, blanket bog, heathland (an amalgamation of wet heath, dry heath and unclassified heath), improved grassland, rough grassland, montane, broadleaf woodland, coniferous woodland, mixed woodland and, in addition, we created a generic “woodland” category (an amalgamation of the broadleaf, coniferous, and mixed woodland categories); Table 1. For analysis we used the proportion of land area around each farm (within a 5 km radius) that contained each land cover type. A distance of 5 km was chosen because 90% of the farms held their sheep within 5 km of the farmhouse. These habitat values were then arc-sin square-root transformed as they were proportions.

Sheep and cattle density data were obtained from the national agricultural census data (AgCensus), available at the Parish level, at a 2 km^2^ grid resolution.

Approximate red deer densities were derived from Deer Commission for Scotland (now Scottish Natural Heritage) count data, based on dedicated observer counts of individual deer from the ground or air, Krigged to a 2 km grid. These data are the best (indeed only) quantitative deer data available, but have several caveats. For example, red deer counts were conducted where the 44 Deer Management Groups areas are, but these cover only around 75–80% of Scotland. Furthermore, the counts for different areas were not always conducted at the same time but, instead, staggered between 2000 and 2006. Therefore, given the level of error in these data, we consider any positive results linking deer density to LIV seroprevalence in sheep to be highly conservative.

*Intersect Point* tool was used in ArcMap v9.3 (ESRI, 2008) to extract the values of all environmental parameters at the locations of each of the 125 sheep farms from a set of raster and vector maps of environmental data.

### Statistical Analysis

To test for environmental variables (habitat, climate and tick hosts) associated with the sero-prevalence of louping ill virus among sheep farms we used general linear mixed models using the glimmix procedure in SAS Version 9.1. The response variable was seroprevalence for each farm expressed as the number of positive serum samples divided by the number of samples assayed for each farm. This is more powerful than using merely a single figure for the proportion of positives because it allows the model to take into account the number of samples taken, which varied from 26 to 29. A binomial distribution was specified. The data distribution was over-dispersed and zero-inflated (i.e., a disproportionate number of zero counts than expected from a Poisson distribution), which is commonly found with disease prevalence data such as these. Therefore, each data point, i.e., individual farm, was entered as a random effect in the model (33) as a way of increasing flexibility of model fit to allow for such overdispersion.

Because of the large number of potential climate-related explanatory variables (Table 1) and because many of the climate variables are inter-related, a variable selection procedure was conducted. All related variables within a climate category (temperature or precipitation) were entered into the model separately and we chose which (within each category) had the strongest individual effect in terms of F and *P*-values for the variable and model AIC. We also then entered all climate variables within a category into the model simultaneously to identify which had the strongest overall effect in terms of F and *P*-values and change in AIC. This variable selection procedure selected annual growing degree days (day-by-day sum, over a year, of the mean number of degrees by which the air temperature is more than 5.5°C) from the temperature-related variables, and the number of dry days from the precipitation-related variables.

**Figure 1:**
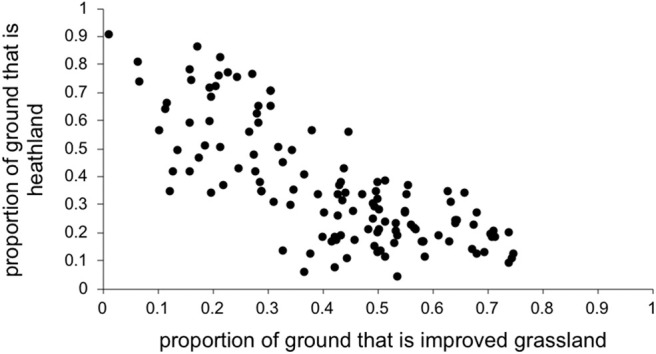
Improved grassland covaried with heathland, so each was entered into the model separately to obtain outputs.

Thus, we entered the following selected explanatory variables as fixed effects into the model: easting and northing (to take into account any spatial autocorrelation), time of year that bloods were sampled (December-March, April-July, August-November), year of blood sampling, estimated deer density, estimated sheep density, growing degree days, dry days, and the proportion of land cover that was each habitat category listed in Table 1. Because the habitat proportions are not independent of each other (e.g., if there is 90% heathland there cannot be more than 10% of any other habitat; see Figure 1) we entered each habitat category separately into the model, i.e., the model never had more than one habitat category at once.

**Figure 2:**
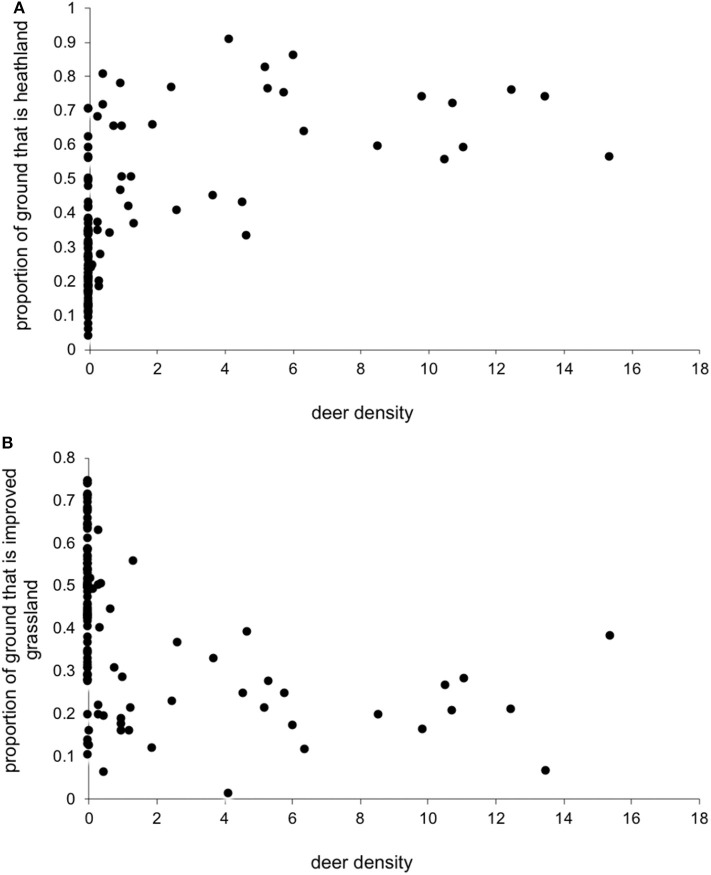
Estimated red deer density (Km^−2^) and the proportion of land cover that was **(A)** heathland and **(B)** improved grassland within a 5 Km radius of each farm location.

We had expected deer density to covary positively with the proportion of heathland. Also, because areas with more heathland have less improved grassland (Figure 1), we expected deer density to negatively covary with improved grassland. However, surprisingly, there was little clear relationship between deer density and the proportion of each of these two key habitat types (Figure 2), so we could include deer simultaneously with each separate habitat in the model.

We conducted a backwards stepwise procedure, whereby we sequentially removed from the model all explanatory variables that did not improve the model: we first removed each variable that had very low significance, i.e., *p* > 0.1, and then checked that removal did not adversely affect model fit (increase AIC). If removal had increased AIC, the variable would have been kept in the model, but this did not occur in our procedure. Because such terms were eliminated from the models, we present test statistics for only those fixed effects that remained in the final model.

## Results

**Figure 3:**
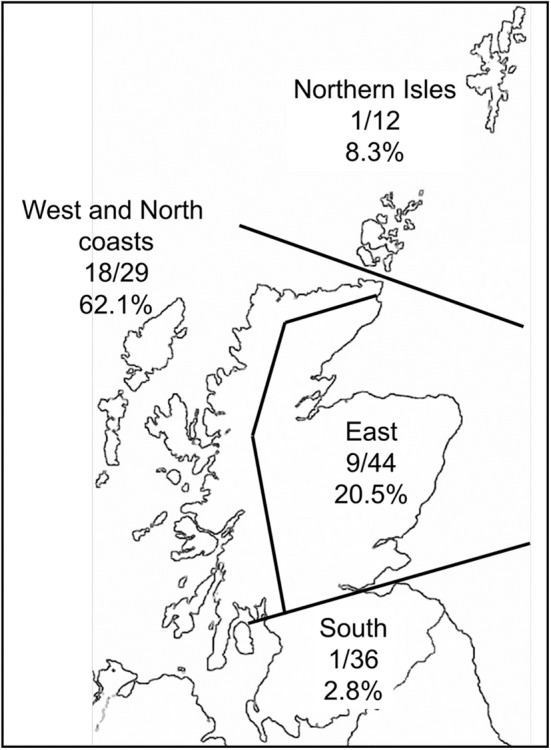
Map of Scotland with the proportion of farms in contrasting areas that had at least one ewe testing seropositive to louping ill virus.

Of the 125 sheep farms sampled, 28 (22.4%) farms contained LIV seropositive sheep (from the 27 individuals per farm blood-sampled). The proportion of positive farms varied greatly over different areas of Scotland (Figure 3), with a much higher proportion along the West and North coasts than in other areas.

**Figure 4:**
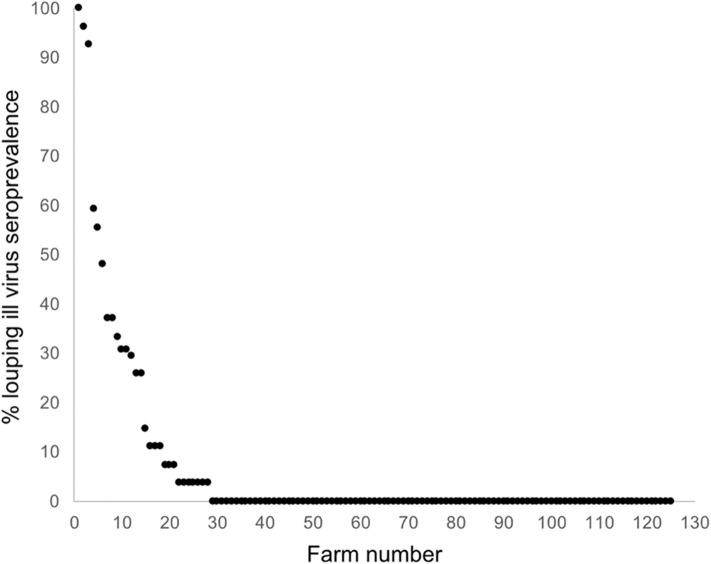
Frequency distribution of LIV seroprevalence across 125 sheep farms randomly stratified around Scotland.

The national average seroprevalence, including farms that did not have LIV, was 6.39% (range 0–100%; median 0%). If only sero-positive farms are considered, the average seroprevalence was 28.52% (range 3.7–100%; median 25.93%). The frequency distribution of within-farm LIV seroprevalence exhibited a negative binomial distribution typical of disease and count data, with most farms having no or low infection, and a small number having very high infection rates (Figure 4).

**Figure 5:**
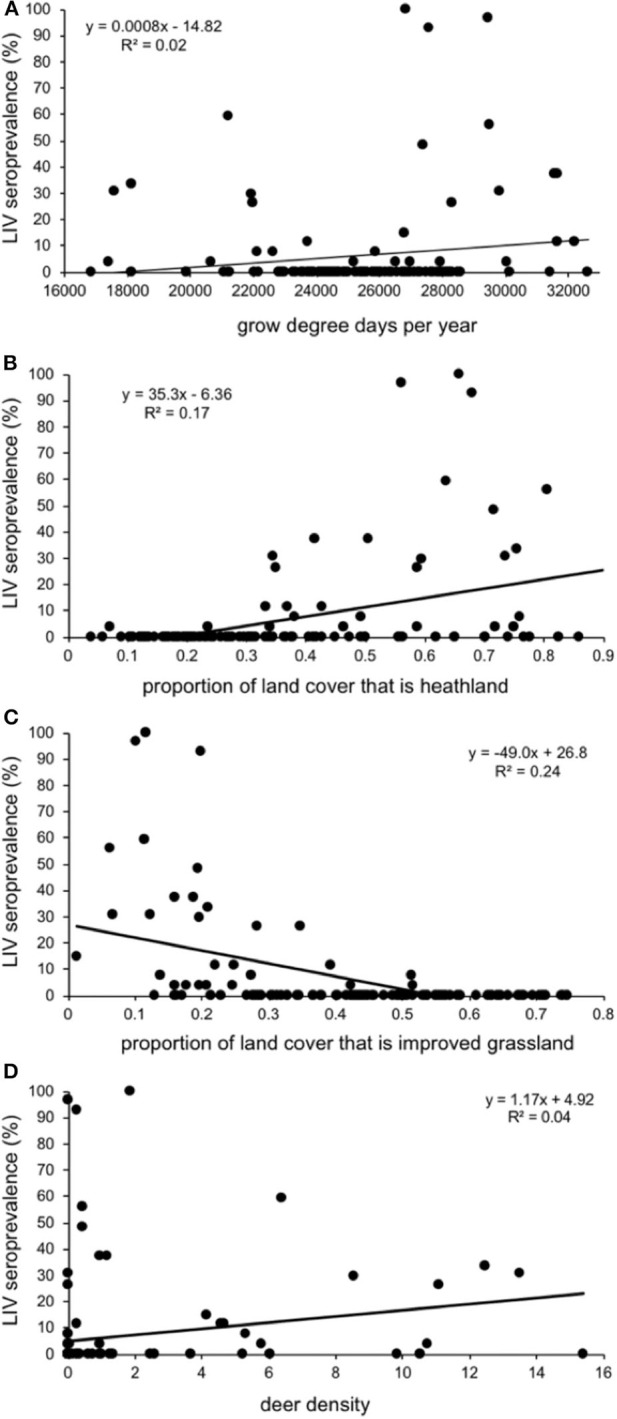
Relationship between seroprevalence of louping ill virus in 125 Scottish sheep farms (% of individuals testing seropositive) and **(A)** growing degree days per year; the proportion of land cover that is **(B)** improved (smooth) grassland and **(C)** heath-dominated moorland; and **(D)** estimated density of red deer in Deer Management Group areas. Each data point represents a sheep farm, and the positions are from the raw unadjusted data, not from the model output.

Sheep farms had higher within-farm LIV seroprevalences (% of ewes within a farm that tested positive) if they were in areas with a warmer climate (more growing degree days per annum), a higher proportion of land that is heath-dominated moorland, a lower proportion of land under improved (smooth) grassland and areas with higher deer densities (Table 2; Figure 5). There was no association between LIV within-flock seroprevalence and the proportion of land cover that was blanket bog, bracken, montane, coarse (rough) grassland or woodland (either broad-leaved, coniferous, mixed or the generic “woodland” category), nor sheep or cattle density, northing, easting, or time of year, or year the blood sample was taken; all these variables were removed from the model during the backwards stepwise procedure.

**Table 2:** Output from the final model identifying environmental parameters that were significantly associated with LIV seroprevalence among sheep farms in Scotland.

	**Estimate**	**SE**	**df**	***F***	***P***
Intercept	−20.606	3.052	101		
Growing degree days	0.000483	0.000105	1, 88	21.23	<0.0001
Deer density	0.276	0.1064	1,83	6.73	0.0112
Heath-dominated moorland	8.0992	1.6500	1,103	24.09	<0.0001
Smooth (improved) grassland	−12.6596	2.4289	1,105	27.16	<0.0001

*Growing degree days are the day-by-day sum (over a year) of the mean number of degrees by which the air temperature is more than 5.5°C. Habitats heath-dominated moorland and smooth (improved grassland) were arcsin square-root transformed as they were proportions. Since they are therefore interdependent they were entered separately into the model*.

## Discussion

We aimed to test the hypothesis that LIV prevalence in sheep farms is influenced by environmental factors, especially those associated with tick abundance and LIV transmission hosts.

In support of our predictions, sheep farms had higher LIV seroprevalences if they were in areas with a warmer climate (more growing degree days per annum). Higher temperatures increase tick interstadial development rate, oviposition rate, egg development rates and tick activity (34–37), and warmer climates (for example, as studied using altitude) have been associated with higher tick abundance (9, 20, 21, 27, 38, 39) and higher risk of tick-borne diseases (examples from Lyme disease risk or *B. burgdorferi* prevalence: (7, 22, 40). While growing degree days is a variable originating from plant growth, it is a measure of warmth, and is particularly relevant to ticks because the plant growing season aligns well with the tick activity season (usually April-October, depending on the area). One potential source of error with the climate parameters is that the blood samples were taken 6 years after the 1971–2000 time period over which the climate data were derived. However, we would not expect the association between climate and tick populations to change over time, i.e., we still expect more ticks (due to higher tick activity and development rates etc.) in areas with a warmer climate irrespective of the year of blood sampling. A further limitation is the spatial scales for some of the climate parameters, especially those variables at the broadest 5 km spatial scale. However, compared to the large, national-scale patterns we were investigating, they proved useful enough for examining our predicted associations with tick-borne disease infection.

As predicted, there were higher LIV seroprevalences among sheep farms in heather moorland which is the characteristic habitat in upland UK and is the habitat most frequented by wildlife hosts that are competent LIV transmitters: red grouse and mountain hares. Upland areas with more heather moorland had less improved grassland (Figure 1). Improved grassland is a habitat more common in the lowlands and more productive farmland areas and was associated in this study with lower LIV seroprevalence. This makes sense, as alternative tick or LIV transmission hosts such as red grouse and mountain hares do not tend to frequent lowland improved grassland habitats and (41) demonstrated fewer ticks on improved pastures compared to upland habitats such as rough hill pastures. Likewise, we expected a strong relationship between deer density and proportion of heathland (positively) and improved grassland (negatively). However, our variable selection procedure found no clear relationship between deer density and the proportion of heathland, nor the proportion of improved grassland (Figure 2), Nonetheless, Figure 2 does indicate that most positive counts of deer are in areas with at least 30% cover of heathland and <50% improved grassland. Including both deer density and % habitat cover in the model simultaneously meant that any effect of deer was over and above the effect of habitat, i.e., the significant effect of deer on LIV seroprevalence was not because there was higher LIV prevalence on heathland. In terms of deducing the mechanism for these effects of habitat and deer on LIV seroprevalence it would have been more informative to include data on densities of red grouse, mountain hares and ticks; however, not enough detailed spatial data were available, hence using habitat as a proxy for these very habitat-specific LIV transmission hosts. More detailed, accurate and extensive deer count data would also be invaluable.

It is particularly interesting to policy and management that we found higher LIV seroprevalences in areas with higher estimated densities of red deer. This was despite the data on red deer being patchy, staggered over several years, and not covering all of Scotland. We therefore consider any result involving a significant effect of deer highly conservative. Thus, although the association between red deer densities and LIV seroprevalence was statistically weak and included a lot of variation, the fact that we found a significant association at all could be indicative of a much stronger association in reality. While red deer do not transmit LIV between ticks (42) deer of various species are often the key drivers of Ixodid tick populations in both Europe (7, 20, 43, 44) and North America (45–50), including red deer in Scotland (10, 19, 21, 24, 40). Because of this, several theoretical models of the LIV system in Scotland predict a key role of deer in the persistence of LIV in ticks and both red grouse and sheep systems (51–54). That deer densities are associated with higher tick abundance and have also been shown in some previous studies to be associated with higher incidence or risk of other tick-borne diseases (both Lyme disease and tick-borne encephalitis: (7, 40, 46, 55, 56), as well as LIV as shown in this study, is highly relevant to policy on disease management. Deer can be managed through various means, most commonly through exclusion from areas using fencing, or by reducing densities through culling. These are common management techniques for the purposes of habitat management for conservation or protection of commercial forestry or crops. Deer management through exclosure fencing and culling have both been found to dramatically control densities of *I. ricinus* ticks in Scotland in both upland heather moorland and forest habitats at a range of spatial scales (10). Reducing deer densities can have impacts not considered in theoretical models, such as increase ground vegetation height (57) and therefore rodent density (58, 59). However, rodents are generally not considered drivers of tick populations, nor competent transmission hosts for LIV (14). It remains possible that reducing deer densities on upland heather moorland could result in an increase in red grouse or mountain hares (LIV transmission hosts), which may potentially dampen the desired reduction of LIV in sheep. However, large-scale field experiments would be needed to test for such unintended consequences, and these would be difficult to achieve in terms of resources, available space and enough replication (e.g., see critique and discussion of a landscape-scale experiment testing the impact of mountain hare control on LIV in grouse: (60–62).

Against our predictions, we did not find a significant association between LIV seroprevalence and the proportion of land cover that was woodland. However, a previous more detailed study of ticks (not LIV) found that (i) distance to woodland and (ii) the proportion of sheep pasture that had tree cover were strong predictors of tick burdens on lambs and tick densities in sheep pastures in Norway (26). One reason for this difference could be potential error with matching our spatial GIS land cover data (5 km buffer zone around the postal address of the farm) with where the sampled sheep actually spent most of their time during the peak tick season in spring/summer. Data from (19) on tick densities on open moorland suggested (non-significantly) higher tick densities only within 50 m from (unfenced) woodland boundaries, and (63) showed striking differences in Lyme disease hazard (the density of ticks infected with *B. burgdorferi*) between woodlands and adjacent open habitats which were often only 50–100 m apart, which suggests that any link between woodlands and tick or tick-borne risk incidence in adjacent open habitats probably operates at a much finer spatial scale than we had access to in this study.

Geographically, the proportion of farms testing seropositive to LIV was much higher along the West and North coasts of Scotland than in other areas. This might be expected given the warm, humid climate which aids tick survival, activity and development. There was low LIV seroprevalence in the Northern Isles (Shetland and Orkney) which is most likely attributed to very low *I. ricinus* tick densities in most areas of these islands (Gilbert unpublished data). This is likely due to a combination of the lack of deer and the colder climate which inhibits tick activity and development. The eastern regions of Scotland had intermediate seroprevalence. These areas, especially Grampian Region, Speyside and Perthshire, have a particularly heterogeneous landscape, from high quality improved grassland for cattle up to high altitude (1,400 m) montane habitats, with extensive forested areas and heather moorland in between. Some of these heathlands have the highest deer, red grouse and mountain hare densities in Scotland. Here, therefore, we would expect a wide spectrum of LIV seroprevalences, which is reflected by the overall intermediate values over the whole region. South of the Central Belt of Scotland there were even lower seroprevalence than the Northern Isles, even with a good sample size of 36 farms. The main habitats are upland rough grasslands and commercial coniferous forests, with some improved grassland for high density livestock grazing. There are deer, and some mountain hares and red grouse present, although not at the densities found in the East region of Scotland. We would therefore expect lower LIV infection rates than in the East region, but it is not clear why the seroprevalence is as extremely low as it is. This could be due to unconsidered factors such as historical movements of infected sheep and warrants further research.

Although exposure of sheep to ticks can be mitigated by acaricide application to the animals (64) alternative measures to reduce exposure are increasingly being sought, such as management of habitats or wild hosts, separation of livestock from tick-infested areas or the application of biological control agents to the pastures (65). By identifying which areas, habitats and environmental conditions pose the greatest LIV risk we can now start to inform policy on the implementation of these alternative approaches, and more efficiently target standard disease control measures to be prioritized in the highest risk areas, periods of time and conditions.

## Data Availability Statement

The datasets generated for this study are available on request to the corresponding author.

## Ethics Statement

The study design was reviewed and approved by Moredun Research Institute.

## Author Contributions

CC and FB initiated and designed the blood collecting sampling strategy as part of a separate study on Ovine pulmonary adenocarcinoma (OPA, also known as jaagsiekte). FB organized farm visits and collected blood samples and KW conducted the LIV serology tests. LG wrote the LIV proposal to Scottish Government, assimilated the separate data sets, analyzed the data and wrote the paper. CC, FB, and KW commented on the manuscript drafts. All authors contributed to the article and approved the submitted version.

## Conflict of Interest

The authors declare that the research was conducted in the absence of any commercial or financial relationships that could be construed as a potential conflict of interest.

## References

[1] Moennig V Houe H Lindberg A. BVD control in Europe: current status and perspectives. Anim Heal Res Rev. (2005) 6:63–74. 10.1079/AHR2005102 16164009

[2] Delahay RJ Cheeseman CL Clifton-Hadley RS Wildlife disease reservoirs: the epidemiology of mycobacterium bovis infection in the Eeuropean badger (meles meles) and other British mammals. Tuberculosis. (2001) 81:43–9. 10.1054/tube.2000.0266 11463223

[3] Naranjo V Gortazar C Vicente J de la Fuente J. Evidence of the role of European wild boar as a reservoir of mycobacterium tuberculosis complex. Vet Microbiol. (2008) 127:1–9. 10.1016/j.vetmic.2007.10.002 18023299

[4] Innocent GT Gilbert L Jones EO McLeod JE Gunn G McKendrick IJ. Combining slaughterhouse surveillance data with cattle tracing scheme and environmental data to quantify environmental risk factors for liver fluke in cattle. Front Vet Sci. (2017) 4:1–12. 10.3389/fvets.2017.00065 28534030 PMC5421147

[5] Bennema SC Ducheyne E Vercruysse J Claerebout E Hendrickx G Charlier J Relative importance of management, meteorological and environmental factors in the spatial distribution of fasciola hepatica in dairy cattle in a temperate climate zone. Int J Parasitol. (2011) 41:225–33. 10.1016/j.ijpara.2010.09.003 20887726

[6] Perret JL Guigoz E Rais O Gern L. Influence of saturation deficit and temperature on ixodes ricinus tick questing activity in a Lyme borreliosis-endemic area (Switzerland). Parasitol Res. (2000) 86:554–7. 10.1007/s004360000209 10935905

[7] Rizzoli A Merler S furlanello C Genchi C. Geographical information systems and bootstrap aggregation (bagging) of tree-based classifiers for lyme disease risk prediction in Trentino, Italian Alps. J Med Entomol. (2002) 39:485–92. 10.1603/0022-2585-39.3.485 12061445

[8] Estrada-Peña A. The relationships between habitat topology, critical scales of connectivity and tick abundance Ixodes ricinus in a heterogeneous landscape in northern Spain. Ecography (Cop). (2003) 26:661–71. 10.1034/j.1600-0587.2003.03530.x14570137

[9] Jouda F Perret J-L Gern L. Ixodes ricinus density, and distribution and prevalence of borrelia burgdorferi sensu lato infection along an altitudinal gradient. J Med Entomol. (2004) 41:162–9. 10.1603/0022-2585-41.2.162 15061274

[10] Gilbert L Maffey GL Ramsay SL Hester AJ. The effect of deer management on the abundance of ixodes ricinus in Scotland. Ecol Appl. (2012) 22:658–67. 10.1890/11-0458.1 22611862

[11] Reid HW Doherty PC. Experimental louping-ill in sheep and lambs. I. Viraemia and the antibody response. J Comp Pathol. (1971) 81:291–8. 10.1016/0021-9975(71)90103-4 5091668

[12] Reid HW Experimental infection of red grouse with louping-ill virus (Flavivirus group). I. The viraemia and antibody response. J Comp Pathol. (1975) 85:223–9. 10.1016/0021-9975(75)90063-8 167055

[13] Gilbert L. Louping ill virus in the UK: a review of the hosts, transmission and ecological consequences of control. Exp Appl Acarol. (2016) 68:363–74. 10.1007/s10493-015-9952-x 26205612

[14] Gilbert L Jones LD Hudson PJ Gould EA Reid HW. Role of small mammals in the persistence of Louping-ill virus: field survey and tick co-feeding studies. Med Vet Entomol. (2000) 14:277–82. 10.1046/j.1365-2915.2000.00236.x 11016435

[15] Laurenson MK Mckendrick IJ Reid HW Challenor R Mathewson GK. Prevalence, spatial distribution and the effect of control measures on louping-ill virus in the forest of Bowland, lancashire. Epidemiol Infect. (2007) 135:963–73. 10.1017/S0950268806007692 17346361 PMC2870653

[16] Newborn D Baines D. Enhanced control of sheep ticks in upland sheep flocks: repercussions for red grouse co-hosts. Med Vet Entomol. (2012) 26:63–9. 10.1111/j.1365-2915.2011.00989.x 22112150

[17] Sheahan BJ Moore M Atkins GJ. The pathogenicity of louping Ill virus for mice and lambs. J Comp Pathol. (2002) 126:137–46. 10.1053/jcpa.2001.0533 11945002

[18] Ogden NH Bown K Horrocks BK Woldehiwet Z Bennett M. Granulocytic ehrlichia infection in Ixodid ticks and mammals in woodlands and uplands of the U.K. Med Vet Entomol. (1998) 12:423–9. 10.1046/j.1365-2915.1998.00133.x 9824827

[19] Ruiz-Fons F Gilbert L. The role of deer as vehicles to move ticks, Ixodes ricinus, between contrasting habitats. Int J Parasitol. (2010) 40:1013–20. 10.1016/j.ijpara.2010.02.006 20211625

[20] Qviller L Risnes-Olsen N Bærum KM Meisingset EL Loe LE Ytrehus B. Landscape level variation in tick abundance relative to seasonal migration in red deer. PLoS ONE. (2013) 8:e71299. 10.1371/journal.pone.0071299 23951125 PMC3739797

[21] Gilbert L. Altitudinal patterns of tick and host abundance: a potential role for climate change in regulating tick-borne diseases? Oecologia. (2010) 162:217–25. 10.1007/s00442-009-1430-x 19685082

[22] Jore S Viljugrein H Hofshagen M Brun-Hansen H Kristoffersen AB Nygård K. Multi-source analysis reveals latitudinal and altitudinal shifts in range of Ixodes ricinus at its northern distribution limit. Parasit Vectors. (2011) 4:84. 10.1186/1756-3305-4-84 21595949 PMC3123645

[23] Halos L Bord S Cotté V Gasqui P Abrial D Barnouin J. Ecological factors characterizing the prevalence of bacterial tick-borne pathogens in ixodes ricinus ticks in pastures and woodlands. Appl Environ Microbiol. (2010) 76:4413–20. 10.1128/AEM.00610-10 20453131 PMC2897445

[24] Gilbert L Can restoration of afforested peatland regulate pests and disease? J Appl Ecol. (2013) 50:1226–33. 10.1111/1365-2664.12141

[25] Vanwambeke SO Van doninck J Artois J Davidson RK Meyfroidt P Jore S. Forest classes and tree cover gradient: tick habitat in encroached areas of southern Norway. Exp Appl Acarol. (2016) 68:375–85. 10.1007/s10493-015-0007-0 26692382

[26] Gilbert L Brunker K Lande U Klingen I Grøva L Environmental risk factors for Ixodes ricinus ticks and their infestation on lambs in a changing ecosystem: implications for tick control and the impact of woodland encroachment on tick-borne disease in livestock. Agric Ecosyst Environ. (2017) 237:265–73. 10.1016/j.agee.2016.12.041

[27] Barandika JF Berriatua E Barral M Juste RA Anda P García-Pérez AL. Risk factors associated with ixodid tick species distributions in the basque region in Spain. Med Vet Entomol. (2006) 20:177–88. 10.1111/j.1365-2915.2006.00619.x 16796614

[28] Clarke DH Casals A. Techniques for hemagglutination and hemagglutination inhibition with arthropod- borne viruses. Am J Trop Med Hyg. (1958) 7:561–73. 10.4269/ajtmh.1958.7.561 13571577

[29] Laurenson MK Hudson PJ McGuire K Thirgood SJ Reid HW. Efficacy of acaricidal tags and pour-on as prophylaxis against ticks and louping-ill in red grouse. Med Vet Entomol. (1997) 11:389–93. 10.1111/j.1365-2915.1997.tb00427.x 9430120

[30] Reid HW Pow I. Antibody response of sheep following administration of louping-ill virus vaccine. Vet Rec. (1995) 136:638–9. 10.1136/vr.136.25.638 7571273

[31] Fuller RM Smith GM Sanderson JM Hill RA Thomson AG The UK Land Cover Map 2000: construction of a parcel-based vector map from satellite images. Cartographic J. (2002) 39:15–25. 10.1179/000870402787288009

[32] Beyer H Hawths Analysis Tools for ArcGIS. (2004) Available online at: http://www.spatialecology.com/htools

[33] Elston DA Moss R Boulinier T Arrowsmith C Lambin X. Analysis of aggregation, a worked example: numbers of ticks on red grouse chicks. Parasitology. (2001) 122:563–9. 10.1017/S0031182001007740 11393830

[34] Macleod J Ixodes ricinus in relation to its physical environment: II. The factors governing survival and activity. Parasitology. (1935) 27:123–44. 10.1017/S0031182000015420

[35] Randolph SE Green RM Hoodless AN Peacey MF. An empirical quantitative framework for the seasonal population dynamics of the tick Ixodes ricinus. Int J Parasitol. (2002) 32:979–89. 10.1016/S0020-7519(02)00030-9 12076627

[36] Gilbert L Aungier J Tomkins JL. Climate of origin affects tick (Ixodes ricinus) host-seeking behavior in response to temperature: implications for resilience to climate change? Ecol Evol. (2014) 4:1186–98. 10.1002/ece3.1014 24772293 PMC3997332

[37] Tomkins JL Aungier J Hazel W Gilbert L. Towards an evolutionary understanding of questing behaviour in the tick Ixodes ricinus. PLoS ONE. (2014) 9:e110028. 10.1371/journal.pone.0110028 25333919 PMC4198204

[38] Cadenas FM Rais O Jouda F Douet V Humair P Moret J. Phenology of *Ixodes ricinus* and Infection with *Borrelia burgdorferi* sensu lato along a north- and south-facing altitudinal gradient on chaumont mountain, Switzerland. J Med Entomol. (2007) 44:683–93. 10.1603/0022-2585 17695026

[39] Burri C Cadenas FM Douet V Moret J Gern L. Ixodes ricinus density and infection prevalence of borrelia burgdorferi sensu lato along a north-facing altitudinal gradient in the rhône valley (Switzerland). Vector-Borne Zoonotic Dis. (2007) 7:50–58. 10.1089/vbz.2006.0569 17417957

[40] James MC Bowman AS Forbes KJ Lewis F McLeod JE Gilbert L. Environmental determinants of Ixodes ricinus ticks and the incidence of borrelia burgdorferi sensu lato, the agent of Lyme borreliosis, in Scotland. Parasitology. (2013) 140:237–46. 10.1017/S003118201200145X 23036286

[41] Milne A. Pasture improvement and the control of sheep tick (Ixodes Ricinus L.). Ann Appl Biol. (1948) 35:369–78. 10.1111/j.1744-7348.1948.tb07381.x 18101398

[42] Jones LD Gaunt M Hails RS Laurenson K Hudson PJ Reid H. Transmission of louping ill virus between infected and uninfected ticks co-feeding on mountain hares. Med Vet Entomol. (1997) 11:172–6. 10.1111/j.1365-2915.1997.tb00309.x 9226648

[43] Gray JS Kahl O JC SJ. Studies on the ecology of lyme disease in a deer forest in county galway, Ireland. J Med Entomol. (1992) 29:915–20. 10.1093/jmedent/29.6.915 1460628

[44] Estrada-Peña A De La Fuente J Ostfeld RS Cabezas-Cruz A. Interactions between tick and transmitted pathogens evolved to minimise competition through nested and coherent networks. Sci Rep. (2015) 5:10361. 10.1038/srep10361 25993662 PMC4438610

[45] Deblinger RD Wilson ML Rimmer DW Spielman A. Reduced abundance of immature Ixodes dammini (Acari: Ixodidae) following incremental removal of deer. J Med Entomol. (1993) 30:144–50. 10.1093/jmedent/30.1.144 8433321

[46] Kilpatrick HJ Labonte AM Stafford KC. The relationship between deer density, tick abundance, and human cases of lyme disease in a residential community. J Med Entomol. (2014) 51:777–84. 10.1603/ME13232 25118409

[47] Werden L Barker IK Bowman J Gonzales EK Leighton PA Lindsay LR. Geography, deer, and host biodiversity shape the pattern of lyme disease emergence in the thousand islands archipelago of Ontario, Canada. PLoS ONE. (2014) 9:e85640. 10.1371/journal.pone.0085640 24416435 PMC3887107

[48] Stafford KC Denicola AJ Kilpatrick HJ. Reduced abundance of ixodes scapularis (acari: ixodidae) and the tick parasitoid ixodiphagus hookeri (hymenoptera: encyrtidae) with reduction of white-tailed deer. J Med Entomol. (2003) 40:642–52. 10.1603/0022-2585-40.5.642 14596277

[49] Rand PW Lubelczyk C Lavigne GR Elias S Holman MS Lacombe EH. Deer density and the abundance of ixodes scapularis (acari: ixodidae). J Med Entomol. (2003) 40:179–84. 10.1603/0022-2585-40.2.179 12693846

[50] Wilson ML Ducey AM Litwin TS GT S. Microgeographic distribution of immature Ixodes dammini ticks correlated with that of deer. Med Vet Entomol. (1990) 4:151–9. 10.1111/j.1365-2915.1990.tb00273.x 2132979

[51] Gilbert L Norman R Laurenson KM Reid HW Hudson PJ Disease persistence and apparent competition in a three-host community: an empirical and analytical study of large-scale, wild populations. J Anim Ecol. (2001) 70:1053–61. 10.1046/j.0021-8790.2001.00558.x

[52] Jones EO Webb SD Ruiz-Fons FJ Albon S Gilbert L The effect of landscape heterogeneity and host movement on a tick-borne pathogen. Theor Ecol. (2011) 4:435–48. 10.1007/s12080-010-0087-8

[53] Porter R Norman R Gilbert L Controlling tick-borne diseases through domestic animal management: a theoretical approach. Theor Ecol. (2011) 4:321–39. 10.1007/s12080-010-0080-2

[54] Porter R Norman RA Gilbert L. A model to test how ticks and louping ill virus can be controlled by treating red grouse with acaricide. Med Vet Entomol. (2013) 27:237–46. 10.1111/j.1365-2915.2012.01047.x 23088727

[55] Garnett JM Connally NP Stafford KC Cartter ML. Evaluation of deer-targeted interventions on lyme disease incidence in connecticut. Public Health Rep. (2011) 126:446–54. 10.1177/003335491112600321 21553675 PMC3072871

[56] Mysterud A Easterday WR Stigum VM Aas AB Meisingset EL Viljugrein H. Contrasting emergence of lyme disease across ecosystems. Nat Commun. (2016) 7:11882. 10.1038/ncomms11882 27306947 PMC4912636

[57] Buesching CD Newman C Jones JT Macdonald DW Testing the effects of deer grazing on two woodland rodents, bankvoles and woodmice. Basic Appl Ecol. (2011) 12:207–14. 10.1016/j.baae.2011.02.007

[58] Flowerdew JR Ellwood SA Impacts of woodland deer on small mammal ecology. Forestry. (2001) 74:277–87. 10.1093/forestry/74.3.277

[59] van Wieren SE Bakker JP The impact of browsing and grazing herbivores on biodiversity. In: Gordon IJ Prins HHT, editors. The Ecology of Browsing and Grazing. Berlin: Springer (2008). p. 328 10.1007/978-3-540-72422-3_10

[60] Laurenson MK Norman RA Gilbert L Reid HW Hudson PJ Identifying disease reservoirs in complex systems: mountain hares as reservoirs of ticks and louping-ill virus, pathogens of red grouse. J Anim Ecol. (2003) 72:177–85. 10.1046/j.1365-2656.2003.00688.x

[61] Laurenson MK Norman RA Gilbert L Reid HW Hudson PJ Mountain hares, louping-ill, red grouse and harvesting: complex interactions but few data. J Anim Ecol. (2004) 73:811–13. 10.1111/j.0021-8790.2004.00851.x

[62] Cope DR Iason GR Gordon IJ Disease reservoirs in complex systems: a comment on recent work by Laurenson. J Anim Ecol. (2004) 73:807–10. 10.1111/j.0021-8790.2004.00850.x

[63] Gilbert L. How landscapes shape Lyme borreliosis risk. In: Braks MAH van Wieren SE Takken W Sprong H, editors. Ecology and Control of Vector-Borne Diseases. Wageningen: Wageningen Academic Publishers. p. 462. 10.3920/978-90-8686-838-4_11

[64] George JE Pound JM Davey RB Acaricides for controlling ticks on cattle and the problem of acaricide resistance. In: Bowman AS Nuttall PA, editos. Ticks: Biology, Disease and Control. Cambridge, UK: Cambridge University Press p. 408–423. 10.1017/CBO9780511551802.019

[65] Klingen I van Duijvendijk G Biological control of the tick Ixodes ricinus by pathogens and invertebrates. In: Braks MAH van Wieren SE Takken W Sprong H, editors. Ecology and Control of Vector-Borne Diseases. Wageningen: Wageningen Academic Publishers p. 462 10.3920/978-90-8686-838-4_20

